# The ability of SAMHD1-deficient monocytes to trigger the Type I IFN response depends on cGAS and mitochondrial DNA

**DOI:** 10.1016/j.jbc.2025.110430

**Published:** 2025-06-26

**Authors:** Jesse Rabinowitz, Isabelle K. Vila, Charlotte Luchsinger, Cinzia Bertelli, Moritz Schüssler, Clara Taffoni, Bin Cui, Annie Zhi Dai, Mohammad M. Rashid, William J. Cisneros, Daphne Cornish, Juan Redondo, Kathryn A. Jackson-Jones, Lacy M. Simons, Ramon Lorenzo-Redondo, Nadine Laguette, Judd F. Hultquist, Felipe Diaz-Griffero

**Affiliations:** 1 Department of Microbiology and Immunology, Albert Einstein College of Medicine, Bronx, New York, USA; 2 IGMM, Université de Montpellier, CNRS, Montpellier, France; 3 Division of Infectious Diseases, Department of Medicine, Northwestern University Feinberg School of Medicine, Chicago, Illinois, USA; 4 Center for Pathogen Genomics and Microbial Evolution, Institute for Global Health, Northwestern University Feinberg School of Medicine, Chicago, Illinois, USA

**Keywords:** Aicardi–Goutières syndrome, cGAS, type I IFN response, mitochondria, THP-1

## Abstract

In humans, mutations in sterile α motif and histidine-aspartate domain–containing protein 1 (*SAMHD1*) lead to the development of a type I interferonopathy known as Aicardi–Goutières syndrome (AGS). AGS can present with a variety of severe phenotypes in patients, and a hallmark of this disease is chronic activation of type I interferon (IFN) signaling. However, the mechanism through which type I IFN signaling is activated in the absence of functional SAMHD1 is not known. Here, we investigated the molecular pathways that lead to type I IFN signaling activation in the absence of SAMHD1. Our investigations revealed that chronic activation of type I IFN signaling in *SAMHD1*-knockout (KO) monocytes is cyclic GMP–AMP synthase (cGAS)-dependent. Analysis of other nucleic acid sensors showed that type I IFN signaling in *SAMHD1*-KO cells is not dependent on melanoma differentiation-associated protein 5 (MDA5) or retinoic acid–inducible gene I (RIG-I). In agreement with our observation that type I IFN signaling is dependent on cGAS, two inhibitors of the cGAS–stimulator of IFN genes pathway, G140 and H151, effectively prevented type I IFN activation in *SAMHD1*-KO monocytes. We also found that type I IFN signaling in *SAMHD1*-KO monocytes is dependent on type I IFN receptor expression. Further exploration revealed mitochondrial malfunction in SAMHD1-KO monocytes that is likely to leak mitochondrial components into the cytoplasm. Overall, our work suggests that genetic knock out of SAMHD1 leads to mitochondrial disfunction, resulting in the presence of mitochondrial DNA in the cytoplasm, which triggers cGAS and the type I IFN response.

In humans, the 615-amino-acid sterile α motif (SAM) and histidine-aspartate domain (HD)-containing protein 1 (SAMHD1) is a potent HIV-1 restriction factor in non-cycling cells, such as macrophages, dendritic cells, and resting CD4^+^ T cells (1, 2, 3, 4, 5, 6, 7, 8, 9). The SAM domain regulates binding with other SAM domain–containing proteins and with DNA (10). The HD domain has (d)GTP-regulated dNTPase activity (11, 12, 13, 14, 15, 16) and is required for SAMHD1 to bind nucleic acids (17, 18, 19, 20, 21). The dNTPase activity of SAMHD1 correlates with and is necessary but not sufficient for the restriction of HIV-1 replication (11, 13, 14, 22, 23, 24). HIV-1 restriction activity also appears to correlate with the phosphorylation state of threonine 592 in SAMHD1 (22, 23).

Germline mutations in *SAMHD1* are responsible for a rare, inheritable, auto-inflammatory disease, Aicardi–Goutières syndrome (AGS), characterized by the strong, unresolved activation of type I interferon (IFN) signaling. Individuals with AGS develop progressive neurological damage, often resulting in death before 20 years of age (25, 26). In addition to *SAMHD1*, the AGS phenotype has been associated with mutations in six other genes (25), all of which encode proteins involved in nucleic acid metabolism: 3′ repair exonuclease 1 (*TREX1*), *RNAseH2A*, *RNAseH2B*, *RNAseH2C*, adenosine deaminase acting on RNA1 (*ADAR1*), and IFN induced with helicase C domain 1 (*IFIH1*) (25). The best-studied of these genes is *TREX1,* and loss-of-function *TREX1* mutations result in the cytosolic accumulation of DNA derived from endogenous retroelements (27). DNA accumulation in *TREX1*-deficient cells triggers constitutive type I IFN responses (27). *SAMHD1*-KO THP-1 cells also autonomously trigger type I IFN responses (28), possibly due to accumulating nucleic acids activating nucleic acids sensors, such as cyclic GMP–AMP synthase (cGAS), retinoic acid–inducible gene I (RIG-I), or melanoma differentiation-associated protein 5 (MDA5) (29, 30).

Upon binding with cytosolic double-stranded (ds) DNA or DNA–RNA hybrids, cGAS produces a secondary messenger, 2′-3′-cyclic GMP–AMP (cGAMP) (31). cGAMP interacts with and activates stimulator of IFN genes (STING) (31), inducing STING trafficking from the endoplasmic reticulum to the Golgi complex, where it undergoes palmitoylation, recruits tank-binding kinase 1 (TBK1), and contributes to a signaling cascade that activates interferon regulatory factor 3 (IRF3). IRF3 translocates into the nucleus, where it induces the expression of type I IFN (31). Similarly, RIG-I binding with short dsRNA or single-stranded (ss) RNA bearing 5′ phosphates or MDA5 binding with long dsRNA causes these sensors to interact with mitochondrial antiviral signaling, which subsequently activates IRF3. Type I IFNs are secreted into the extracellular compartment, where they bind the Type I IFN receptor (IFNAR). This subsequently activates the Janus kinase (JAK), which induces the transcription of IFN-stimulated genes (ISGs).

We previously used the lentiviral vector–mediated delivery of the CRISPR/Cas9 system to achieve *SAMHD1* knockout (KO) in human monocytes, which resulted in the upregulation of type I IFN responses and ISG expression, including the potent HIV restriction factor myxovirus protein B (MxB) (28). However, type I IFN response activation in *SAMHD1*-KO cells appears to be cell type– and cell line–specific. Although the investigators that initially linked SAMHD1 function to DNA repair observed type I IFN response activation in *SAMHD1*-depleted HeLa cells (32), other investigators reported that *SAMHD1* depletion from HeLa cells did not trigger the Type I IFN response (28, 33). *SAMHD1*-depletion activates the type I IFN response in THP-1 cells (28, 33) but not in human fibrosarcoma HT1080 cells or human keratinocyte HaCaT cells (this work).

Here, we investigated the cellular pathways underlying the type I IFN response activation in SAMHD1-KO monocytic THP-1 cells. Similar to the lentiviral-mediated delivery of the CRISPR/Cas9 system (28, 33), the transient transfection of a guide RNA (gRNA) targeting *SAMHD1* together with the Cas9 protein knocked out SAMHD1 expression and induced an active type I IFN response. We further found that cGAS, but not MDA5 or RIG-I, is essential for the activation of the type I IFN response in *SAMHD1*-KO THP-1 cells. In agreement with our finding that the cGAS–STING pathway activates the type I IFN response in *SAMHD1*-KO THP-1 cells, we found that specific inhibitors targeting the cGAS–STING pathway, G140 and H151, could effectively prevent type I IFN activation in *SAMHD1*-KO THP-1 cells. Furthermore, the type I IFN receptor (IFNAR) signaling is necessary for the type I IFN activation observed in *SAMHD1*-KO THP-1 cells. Thus, our data reveal that cGAS is required for the type I IFN response in *SAMHD1*-KO monocytes, suggesting that inhibition of cGAS–STING signaling could prevent chronic type I IFN signaling in AGS and potentially ameliorate associated symptoms.

## Results

### *SAMHD1*-KO THP-1 cells show increased expression of the ISG MxB and SIGLEC-1

**Figure 1:**
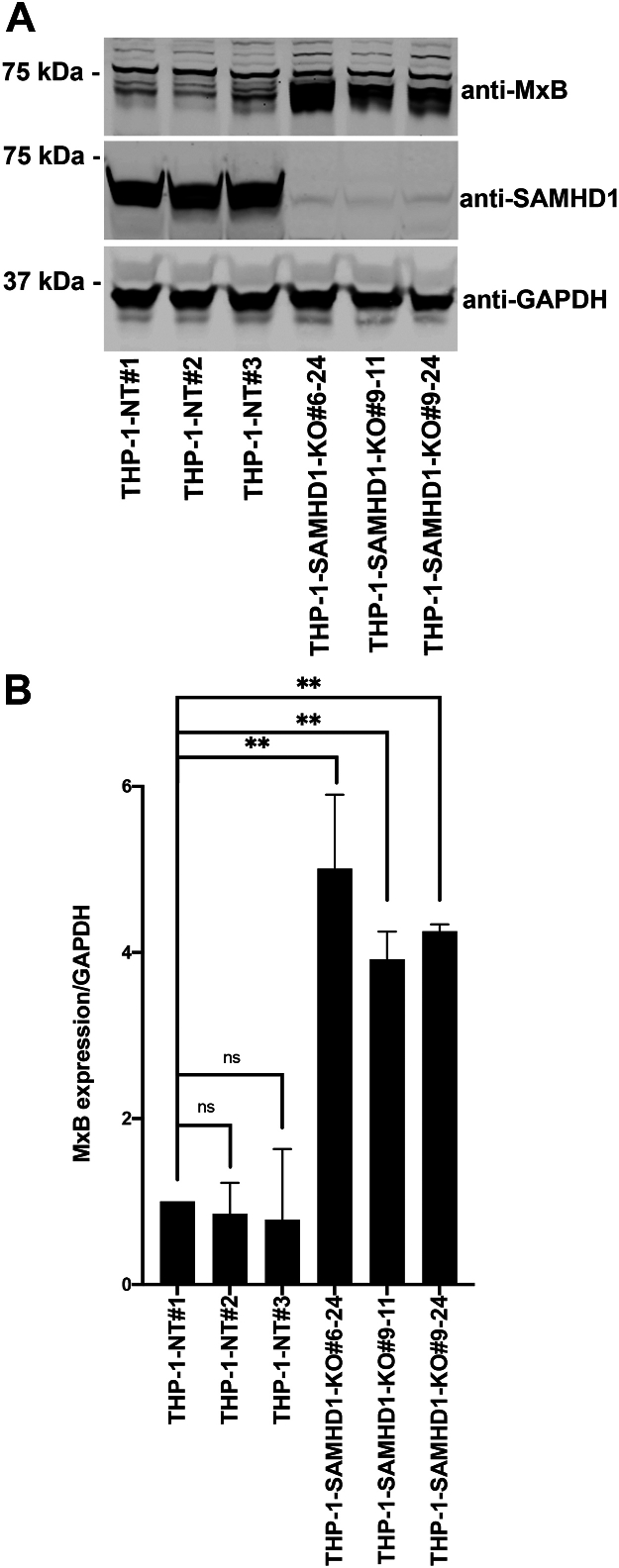

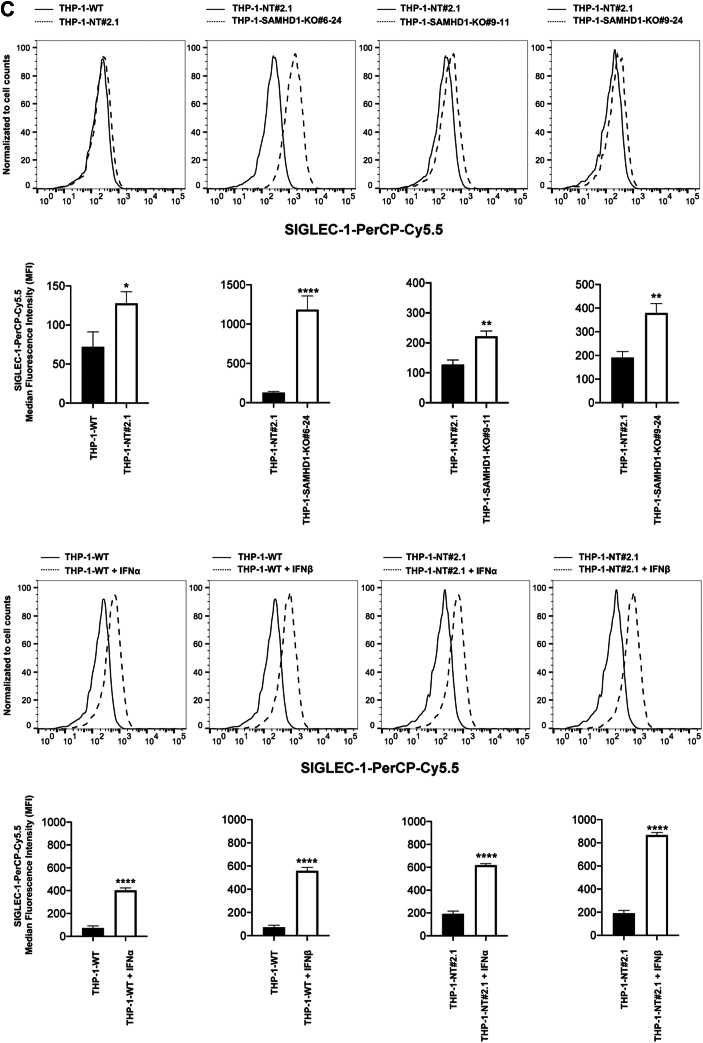

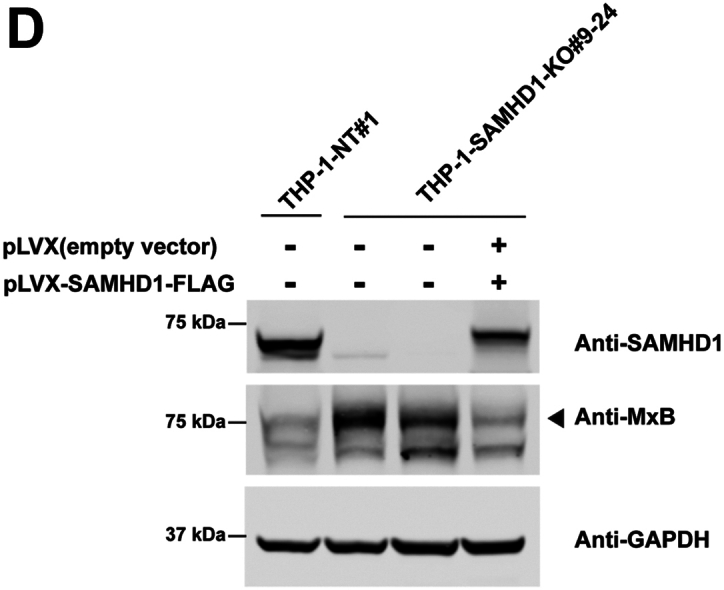
**THP-1 cells with stable *SAMHD1* knockout show increased expression of the interferon-stimulated gene MxB.***A*, THP-1 monocytes were transfected with Cas9 and either sterile α motif and histidine-aspartate domain–containing protein 1 (*SAMHD1*)-targeting guide RNA (gRNA) or non-targeting gRNA by electroporation. Single-cell clones were generated from larger pools of transfected cells. Three separate single-cell *SAMHD1*-knockout (KO) clones (6-24, 9-11, and 9-24) were identified by Western blot using antibodies against SAMHD1. Membranes were subsequently probed with antibodies against MxB. As a loading control, samples were probed with antibodies against GAPDH. Experiments were repeated twice, and a representative example is shown. *B*, quantification of the MxB band intensity normalized against the GAPDH band intensity. Data represent the mean and standard deviation for two independent experiments. Statistical analysis was performed using one-way analysis of variance. ∗∗, *p* < 0.01; ns, not significant. *C*, THP-1 *SAMHD1*-KO cells were analyzed for the surface expression of SIGLEC-1, an interferon activated gene, by flow cytometry using specific antibodies against SIGLEC-1 conjugated to Cy5.5. As control the indicated cells were treated with 1000U/ml of IFNα or IFNβ for 24 h. The Mean Fluoresce Intensity is shown for three independent experiments with standard deviation is shown. Statistical analysis was performed using one-way analysis of variance. ∗, *p* < 0.01; ∗∗∗∗, *p* < 0.0001. *D*, THP-1 SAMHD1 knockout cells were transduced with a lentiviral vector expressing FLAG-tagged SAMHD1 or an empty vector (pLVX) as a control. Protein expression was assessed by Western blot using antibodies against SAMHD1, MxB, and GAPDH. The experiment was performed three times, and a representative blot is shown.

We previously reported that *SAMHD1*-KO human monocytes present with spontaneous type I IFN response activation and upregulated ISG expression (28). In prior experiments, *SAMHD1* KO was achieved using a CRISPR/Cas9 lentiviral vector, resulting in robust long-term expression of Cas9 and *SAMHD1*-targeting gRNA sequences due to their stable integration into the cellular genome. However, robust gRNA expression alone may trigger the type I IFN response (34, 35). To exclude this possibility, we attempted to knock out *SAMHD1* by electroporating wild-type (WT) THP-1 monocytes with the Cas9 protein and a specific *SAMHD1*-targeting gRNA. Unlike the lentiviral-mediated delivery of the CRISPR/Cas9 system, electroporation does not lead to the stable integration of Cas9 and gRNA sequences into the genome. Single-cell monocyte clones were selected, and SAMHD1 expression was evaluated by Western blotting using antibodies against SAMHD1. As shown in Figure 1*A*, the single-cell THP-1 clones 6 to 24, 9 to 11, and 9 to 24 lacked SAMHD1 expression. As a control, we selected single-cell THP-1 clones electroporated with a non-targeting (NT) gRNA (Fig. 1*A*). In agreement with our previous results (28), single-cell THP-1 clones lacking SAMHD1 expression showed upregulated expression of the ISG MxB compared with single-cell THP-1 clones electroporated with the NT gRNA. Quantification of three independent experiments revealed that the level of MxB expression in *SAMHD1*-KO monocytes was 4–6-fold the level in control monocytes (Fig. 1*B*). These results confirm our previous observation that *SAMHD1-*KO monocytes present with spontaneous type I IFN response activity and demonstrate that *SAMHD1* KO can be achieved using a less genetically invasive methodology than stable genomic integration *via* viral vector.

We have previously measured other ISGs in THP-1 SAMHD1 KO cells, and showed an increased expression for *IFIT1*, *MxA,* and *IFNβ* (28). To check for additional ISGs in the new THP-1 *SAMHD1*-KO clones, we measured expression of SIGLEC-1(CD69), which is a surface-expressed protein induced by type I IFN. As shown in Figure 1*C*, all SAMHD1-KO THP-1 clones showed a significant increase in surface expression of SIGLEC-1. As expected, we observed an increase in SIGLEC-1 surface expression when WT and non-targeting control THP-1 cells were treated with IFNα or IFNβ (Fig. 1*C*). These results showed that several ISGs and SIGLEC-1 are induced in *SAMHD1*-KO THP-1 cells.

Next, we investigated whether this phenotype is observed in other cell types. To this end, we knocked out SAMHD1 expression in human epithelial HT1080 and keratinocytes HaCaT cells using CRISPR/Cas9. Contrary to THP-1 cells, HT1080 and HaCaT cells that are knockout for SAMHD1 did not induce expression of MxB (Fig. S1).

To confirm that the phenotype observed in SAMHD1 knockout (KO) THP-1 cells was specifically due to the loss of SAMHD1, we reconstituted SAMHD1 expression by transducing the KO cells with a lentiviral vector encoding wild-type SAMHD-FLAG or the empty vector pLVX (Fig. 1*D*). Restoration of SAMHD1 expression rescued the phenotype of wild-type cells, as evidenced by the suppression of Type I interferon IFN-inducible gene MxB. In particular, reconstitution of SAMHD1 expression prevented the activation of the type I IFN-response, indicating that endogenous SAMHD1 plays a critical role in maintaining immune homeostasis.

### Expression of MxB in SAMHD1-KO cells is dependent on cGAS

**Figure 2:**
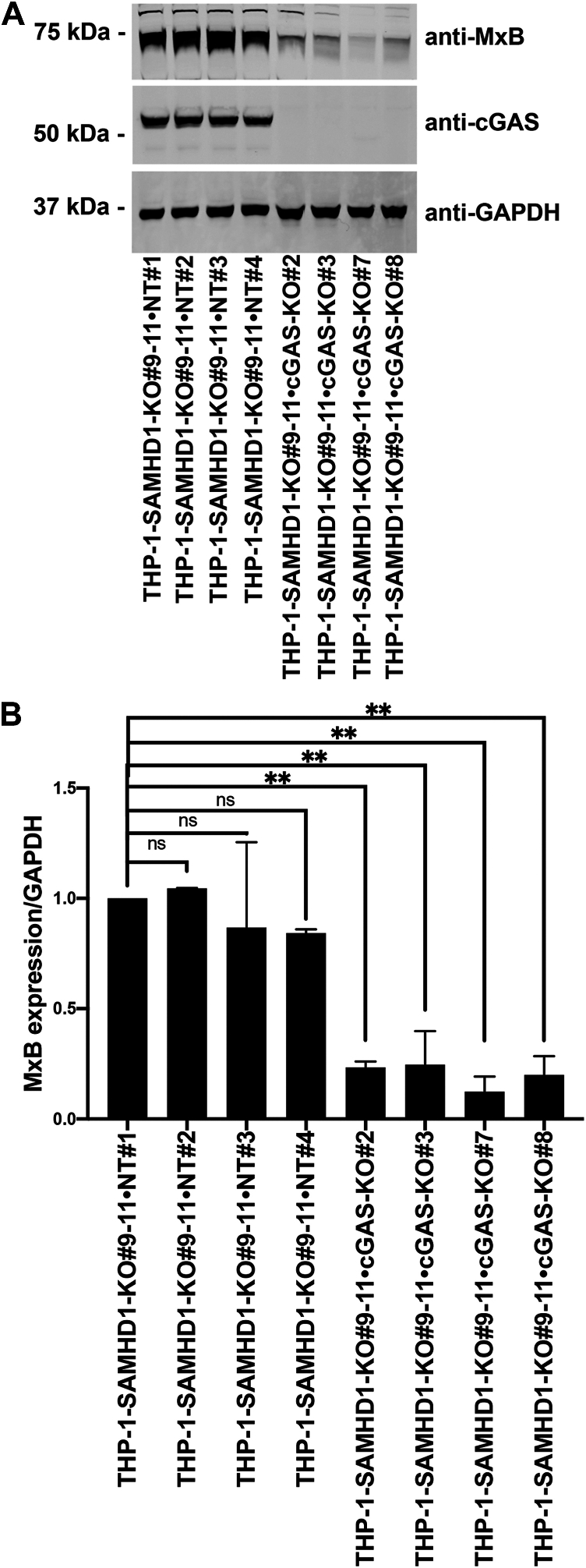

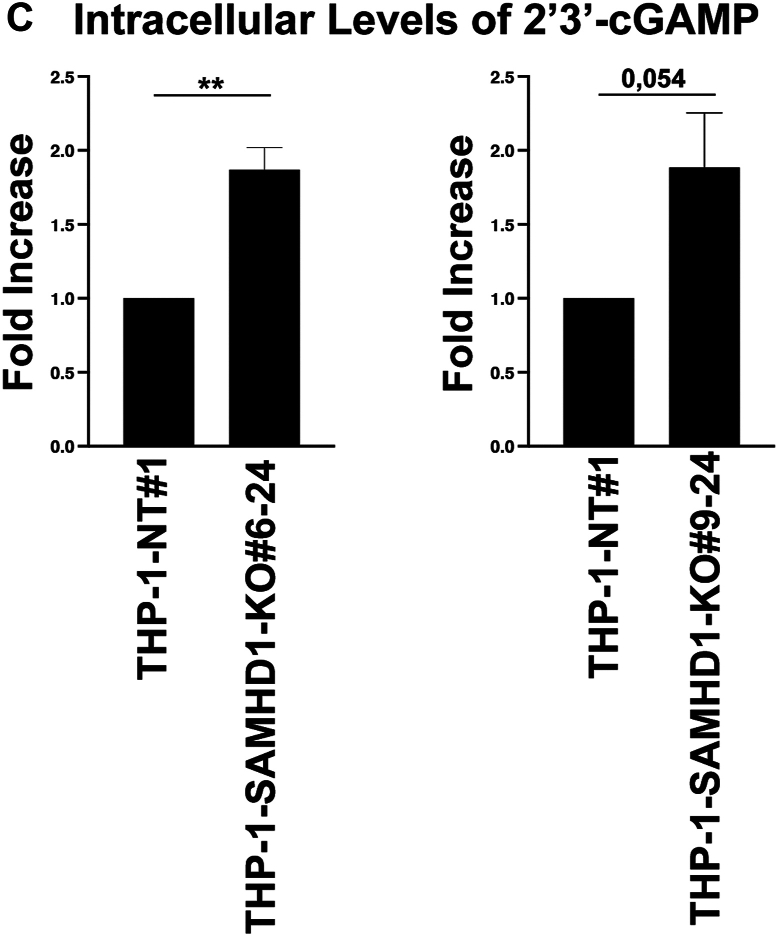
**MxB expression in THP-1–*SAMHD1* KO cells is cGAS-dependent.***A*, THP-1 monocytes with stable sterile α motif and histidine-aspartate domain–containing protein 1 (*SAMHD1*) knockout (KO; THP-1-*SAMHD1*-KO cells) were transfected with Cas9 and either cyclic GMP–AMP synthase (*cGAS*)-specific guide RNA (gRNA) or non-targeting gRNA by electroporation. Single-cell clones were selected from the larger population of transfected cells. Four single-cell clones with simultaneous *SAMHD1* and *cGAS* KO (THP-1–*SAMHD1*-KO•*cGAS*-KO) were identified by Western blot using antibodies against cGAS. Additionally, samples were probed with antibodies against MxB. As a loading control, samples were probed with antibodies against GAPDH. Experiments were repeated twice, and a representative example is shown. *B*, quantification of the MxB band intensity normalized against GAPDH band intensity. Data represent the mean and standard deviation for two independent experiments. Statistical analysis was performed using one-way analysis of variance. ∗∗, *p* < 0.01; ns, not significant. *C*, intracellular 2′3′-cGAMP levels were analyzed in the indicated THP-1-*SAMHD1*-KO and non-targeting (NT) cells. Relative 2′3′-cGAMP levels in the clone cells are normalized to the 2′3′-cGAMP level obtained in the NT cells. Graphs represent means ± standard error from the mean (SEM) of (*A*) n = 3 independent experiments, (*B*) n = 4 independent experiments. Statistical analysis was performed using Student's *t* test. ∗∗, *p* < 0.01; ns, not significant.

We and others have described the ability of SAMHD1 to bind to nucleic acids (17, 18, 19, 20, 21), which may prevent type I IFN response activation by competing with DNA and RNA sensors for nucleic acid–binding. To test this possibility, we assessed whether the type I IFN response observed in *SAMHD1*-KO monocytes was cGAS-dependent, as cGAS triggers the type I IFN response by sensing various types of nucleic acids, including dsDNA, ssDNA, DNA–RNA hybrids, and circular RNA (36). We transfected two independent *SAMHD1*-KO single-cell clones with either NT or cGAS-specific gRNAs using electroporation to generate *SAMHD1* and *cGAS* double-KO THP-1 clones. In the 9 to 11 *SAMHD1*-KO background, the four *cGAS*-KO clones presented with greatly reduced MxB expression compared with the four NT clones (Fig. 2), suggesting that the type I IFN response observed in *SAMHD1*-KO clones is dependent on cGAS activation. In agreement with these findings, we also observed reduced MxB expression in *cGAS*-KO clones compared with NT clones in the 9 to 24 *SAMHD1*-KO background. We conclude that cGAS KO prevents the upregulation of the ISG MxB in *SAMHD1*-KO THP-1 cells.

These results also imply that the cGAS pathway is active in *SAMHD1*-KO THP-1 cells. Activation of the cGAS pathway leads to an increase in the metabolite 2′,3′-cyclic GMP-AMP (cGAMP), which binds and activates STING. To investigate the activity of the cGAS pathway in THP-1 *SAMHD1*-KO cells, we measured the levels of cGAMP. As shown in Figure 2*C*, *SAMHD1*-KO THP-1 cells exhibited elevated levels of cGAMP compared to control cells. This serves as direct evidence that the cGAS pathway is indeed active in *SAMHD1*-KO THP-1 cells.

To functionally test that *SAMHD1* and *cGAS* double-KO THP-1 cells are defective for the cGAS pathway, we transfected double-stranded DNA (dsDNA) in these cells and measured induction of IFNβ and ISG54. As shown in Figure S2, we found that dsDNA was unable to induce expression of IFNβ and ISG54 in double KO cells for SAMHD1 and cGAS when compared to control cells. Similarly, transfection of cGAMP, an STING agonist, which activates the type I IFN response *via* STING, revealed that STING is intact in *SAMHD1* and *cGAS* double-KO THP-1 cells (Fig. S2).

### Pharmacological inhibition of the cGAS–STING pathway prevents MxB upregulation in SAMHD1-KO THP-1 cells

To further examine whether the type I IFN response is cGAS-dependent in *SAMHD1*-KO monocytes, we examined MxB expression in *SAMHD1*-KO THP-1 cells in the presence and absence of inhibitors targeting cGAS and its downstream signaling pathway. G140 is a small-molecule inhibitor that targets cGAS by occupying the reactive ATP- and GTP-binding pocket, preventing cGAMP synthesis and inhibiting downstream pathway activation (37). H151 is a small molecule that targets STING, a downstream component of the cGAS pathway (36), preventing post-translational palmitoylation (38), which is essential for STING clustering and signaling.

**Figure 3:**
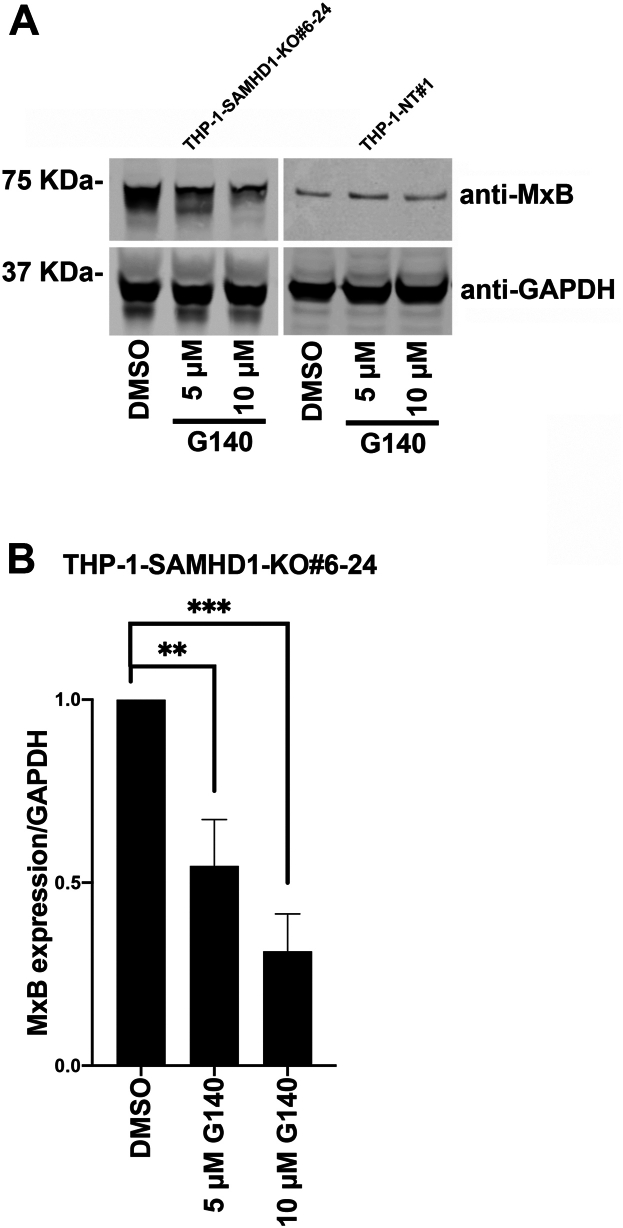
**Pharmacological inhibition of the cGAS pathway using G140 prevents MxB upregulation in THP-1–*SAMHD1*-KO cells.***A*, THP-1 monocytes with stable sterile α motif and histidine-aspartate domain–containing protein 1 (*SAMHD1*) knockout (KO; THP-1-*SAMHD1*-KO cells) were treated with five or 10 μM G140 or an equivalent amount of dimethyl sulfoxide (DMSO). Three days post-treatment, cells were washed with 1× phosphate-buffered saline (PBS) and treated a second time. Three days after the second treatment, cells were lysed and subjected to Western blot analysis using antibodies against MxB and the loading control GAPDH. Experiments were repeated three times, and a representative example is shown. *B*, quantification of MxB band intensity normalized against the GAPDH band intensity. Data represent the mean and standard deviation for three independent experiments. Statistical analysis was performed using one-way analysis of variance. *p* < 0.01; ∗∗∗, *p* < 0.001; ns, not significant.

**Figure 4:**
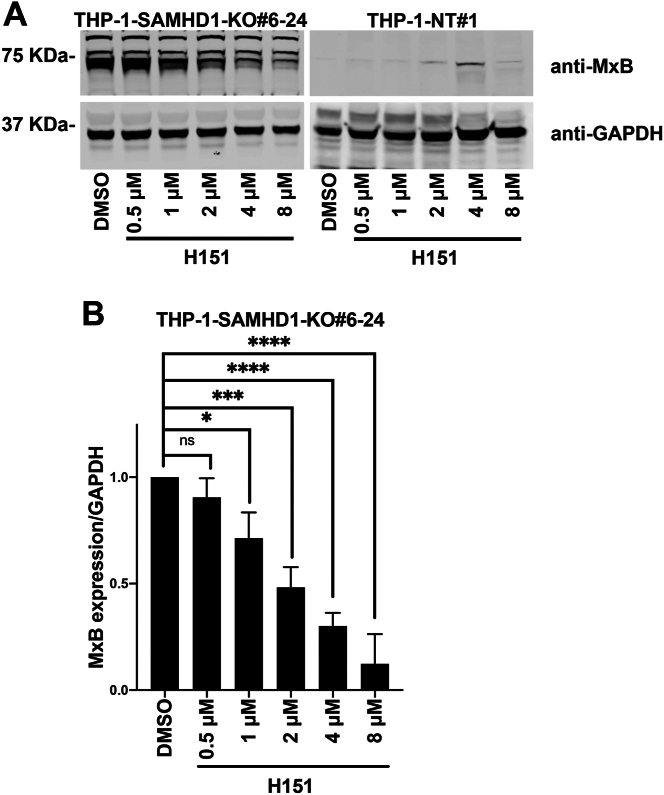
**Pharmacological inhibition of the stimulator of interferon genes pathway using H151 prevents MxB upregulation in THP-1–*SAMHD1*-KO cells.***A*, THP-1 monocytes with stable *SAMHD1* knockout (KO; THP-1-*SAMHD1*-KO cells) were treated with 0.5, 1, 2, 4, or 8 μM H151 or an equivalent amount of dimethyl sulfoxide (DMSO). Three days post-treatment, cells were washed with 1× phosphate-buffered saline (PBS) and treated a second time. Three days after the second treatment, cells were lysed and subjected to Western blot analysis using antibodies against MxB and the loading control GAPDH. Experiments were repeated three times, and a representative example is shown. *B*, quantification of the MxB band intensity normalized against the GAPDH band intensity. Data represent the mean and standard deviation for three independent experiments. Statistical analysis was performed using one-way analysis of variance. ∗, *p* < 0.05; ∗∗∗, *p* < 0.001; ∗∗∗∗, *p* < 0.0001; ns, not significant.

Treatment of the *SAMHD1*-KO THP-1 clone 6 to 24 with five or 10 μM G140 decreased expression of the ISG MxB in a dose-dependent manner compared with dimethyl sulfoxide (DMSO)-treated control cells (Fig. 3). This finding is consistent with our results showing that ISG induction is cGAS-dependent in *SAMHD1*-KO THP-1 cells. Similarly, treatment of the *SAMHD1*-KO THP-1 clone 6 to 24 with various H151 concentrations resulted in a dose-dependent decrease in MxB expression compared with DMSO-treated controls (Fig. 4). Taken together, these results show that the induction of the type I IFN response in *SAMHD1*-KO THP-1 cells is cGAS-dependent.

### KO of cytosolic RNA-sensing proteins MDA5 and RIG-I does not affect MxB expression in *SAMHD1*-KO THP-1 cells

**Figure 5:**
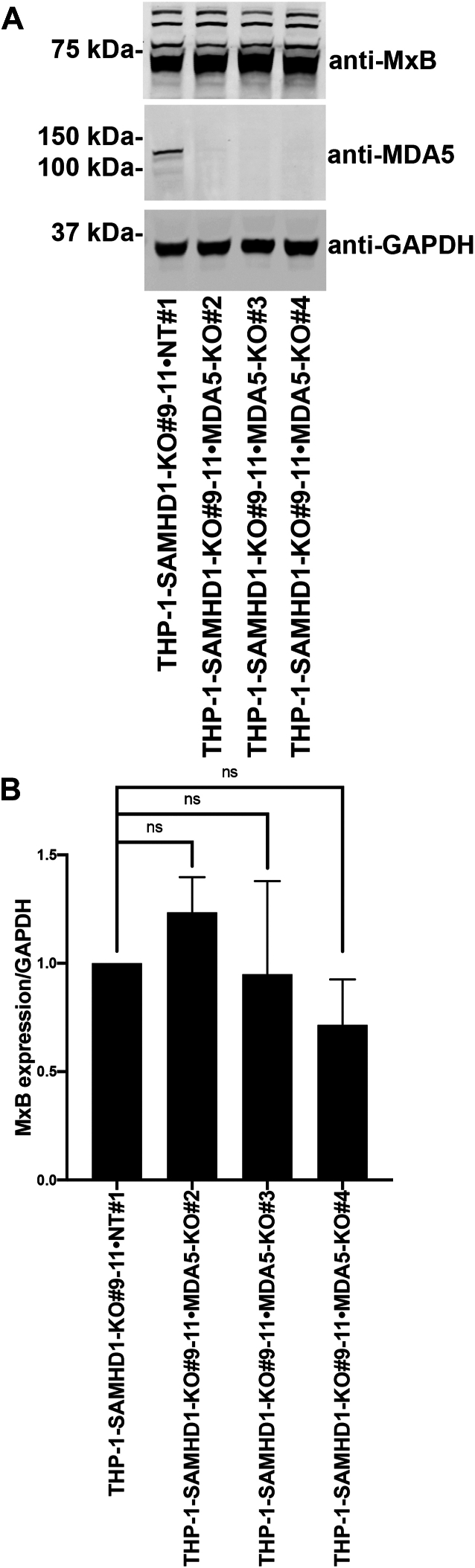
***MDA5* knockout does not prevent MxB expression in THP-1–*SAMHD1*-KO cells.***A*, THP-1 monocytes with *SAMHD1* knockout (KO; THP-1-*SAMHD1*-KO cells) were transfected with Cas9 and either melanoma differentiation-associated protein 5 (*MDA5*)-specific guide RNA (gRNA) or non-targeting gRNA by electroporation. Single-cell clones were isolated from the population of transfected cells. Three single-cell clones with simultaneous *SAMHD1* and *MDA5* KO (THP-1–*SAMHD1*-KO•*MDA5*-KO) were identified by Western blot using antibodies against MDA5. Additionally, samples were probed with antibodies against MxB. Antibodies against GAPDH were used as a loading control. Experiments were repeated twice, and a representative example is shown. *B*, quantification of the MxB band intensity normalized against the GAPDH band intensity. Data represent the mean and standard deviation for two independent experiments.

**Figure 6:**
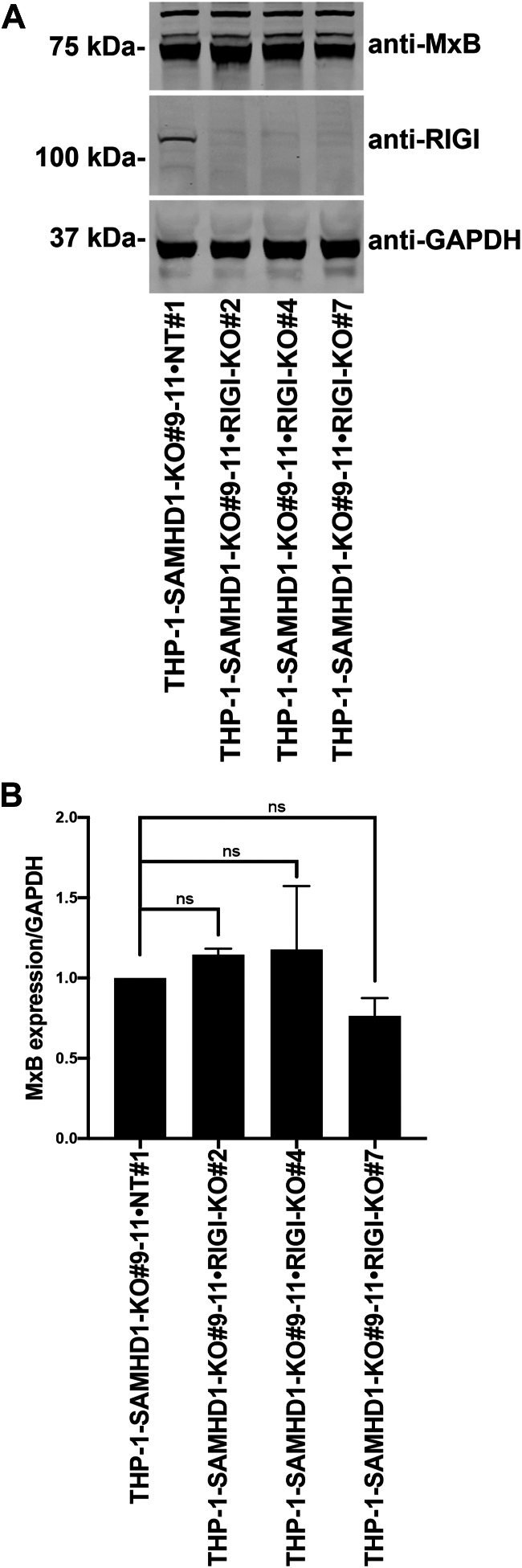
***RIG-I* knockout does not inhibit MxB expression in THP-1–*SAMHD1* KO cells.***A*, THP-1 monocytes with stable *SAMHD1* knockout (KO; THP-1-*SAMHD1*-KO cells) were transfected with Cas9 and either retinoic acid–inducible gene I (*RIG-I*)-specific guide RNA (gRNA) or non-targeting gRNA by electroporation. Single-cell clones were isolated from the larger pool of transfected cells. Three single-cell clones with simultaneous *SAMHD1* and *RIG-I* KO (THP-1–*SAMHD1*-KO•*RIG-I*-KO) were identified by Western blot using antibodies against RIG-I. Additionally, samples were probed with antibodies against MxB. Antibodies against GAPDH were used as a loading control. Experiments were repeated twice, and a representative example is shown. *B*, quantification of the MxB band intensity normalized against the GAPDH band intensity. Data represent the mean and standard deviation for two independent experiments. Statistical analysis was performed using one-way analysis of variance. ns, not significant.

Because SAMHD1 can interact with nucleic acids other than DNA (17, 18, 19, 20, 21), we also evaluated the possibility that RNA might trigger the type I IFN response in *SAMHD1*-KO THP-1 cells. To test this possibility, we generated *MDA5-*and *RIG-I*-KO lines in the *SAMHD1*-KO THP-1 background. In contrast to our findings in *cGAS*-KO cells, *MDA5*- (Fig. 5) and *RIG-I*-KO (Fig. 6) lines in the *SAMHD1*-KO THP-1 background showed no change in MxB expression compared with *SAMHD1* single-KO THP-1 cells. These results suggest that the induction of the type I IFN response in *SAMHD1*-KO THP-1 cells is independent of both MDA5 and RIG-I.

To functionally assess the integrity of the cGAS pathway in SAMHD1 and MDA5 or RIG-I double-KO THP-1 cells, we transfected double-stranded DNA (dsDNA) into these cells and measured the induction of *IFNβ* and *ISG54*. As shown in Figure S2, we showed that SAMHD1 and MDA5 or RIG-I double-KO THP-1 cells were sensitive to dsDNA. These results showed that the cGAS pathway is intact in these double KO cells. In agreement, transfection of cGAMP, an activator of STING, revealed that the STING pathway is active in these cells (Fig. S2).

### Expression of MxB in *SAMHD1*-KO THP-1 cells is dependent upon IFNAR expression

**Figure 7:**
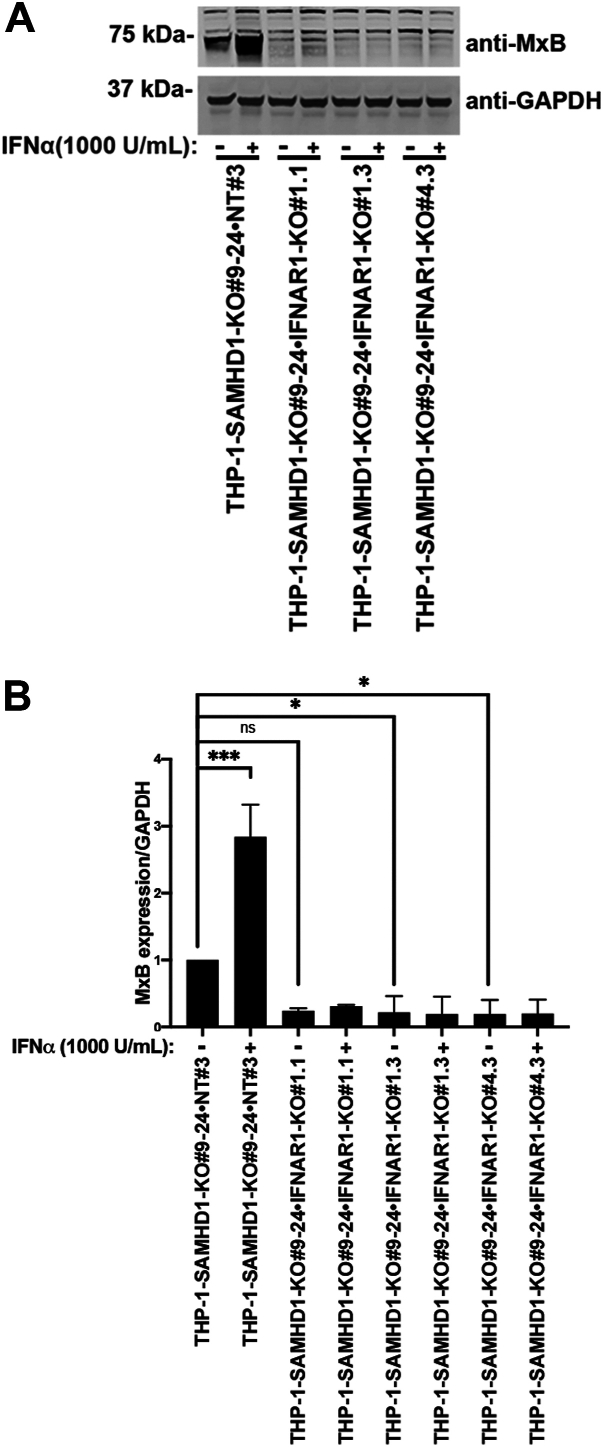
**MxB expression in THP-1–*SAMHD1*-KO cells is dependent upon *IFNAR* expression.***A*, THP-1 monocytes with stable *SAMHD1* knockout (KO; THP-1-*SAMHD1*-KO cells) were transfected with Cas9 and either interferon-alpha (IFNα) receptor (*IFNAR*)-specific guide RNA (gRNA) or non-targeting gRNA by electroporation. Single-cell clones were isolated from the larger population. Three single-cell clones with simultaneous *SAMHD1* and *IFNAR* KO (THP-1–*SAMHD1*-KO•*IFNAR*-KO) were identified by treatment with 1000 U/ml IFNα. Additionally, samples were probed with antibodies against MxB. Antibodies against GAPDH were used as a loading control. Experiments were repeated twice, and a representative example is shown. *B*, quantification of the MxB band intensity normalized against the GAPDH band intensity. Data represent the mean and standard deviation for two independent experiments. Statistical analysis was performed using one-way analysis of variance. ∗, *p* < 0.05; ∗∗∗, *p* < 0.001; ns, not significant.

Our findings showed that the ability of *SAMHD1*-KO THP-1 cells to autonomously induce a type I IFN response is cGAS-dependent. The induction of a type I IFN response and ISG expression supports the production of bioactive IFNs in *SAMHD1*-KO cells. cGAS-dependent type I IFN production results from the detection of cytosolic nucleic acids by cGAS, triggering a signaling cascade that ultimately leads to type I IFN secretion (31) and the promotion of ISG expression through IFNAR stimulation. To test whether the induction of MxB expression in *SAMHD1*-KO THP-1 cells is dependent on IFNAR expression and signal amplification, we generated *SAMHD1* and *IFNAR* double-KO THP-1 cells and measured MxB expression in response to treatment with 1000 U/ml of IFNα (Fig. 7). Our results showed that *SAMHD1* and *IFNAR* double-KO THP-1 cells showed vastly reduced MxB expression compared with *SAMHD1* single-KO THP-1 cells. These results suggest that autonomous activation of the type I IFN response in *SAMHD1*-KO THP-1 cells is dependent on the expression of IFNAR.

### Less active mitochondria are observed in *SAMHD*-1-KO cells

Our investigations suggest that a nucleic acid triggers the Type I IFN response in SAMHD1-KO THP-1 cells; however, the exact nature of this signal remains unknown. Mitochondria harbor various components that can act as damage-associated molecular patterns (DAMPs) when released into the cytoplasm, including N-formylated peptides, hypomethylated mitochondrial DNA (mtDNA), mitochondrial RNA–DNA hybrids, and ATP (39, 40). It is well established that cytoplasmic mtDNA activates the cGAS–STING pathway (41, 42, 43).

**Figure 8:**
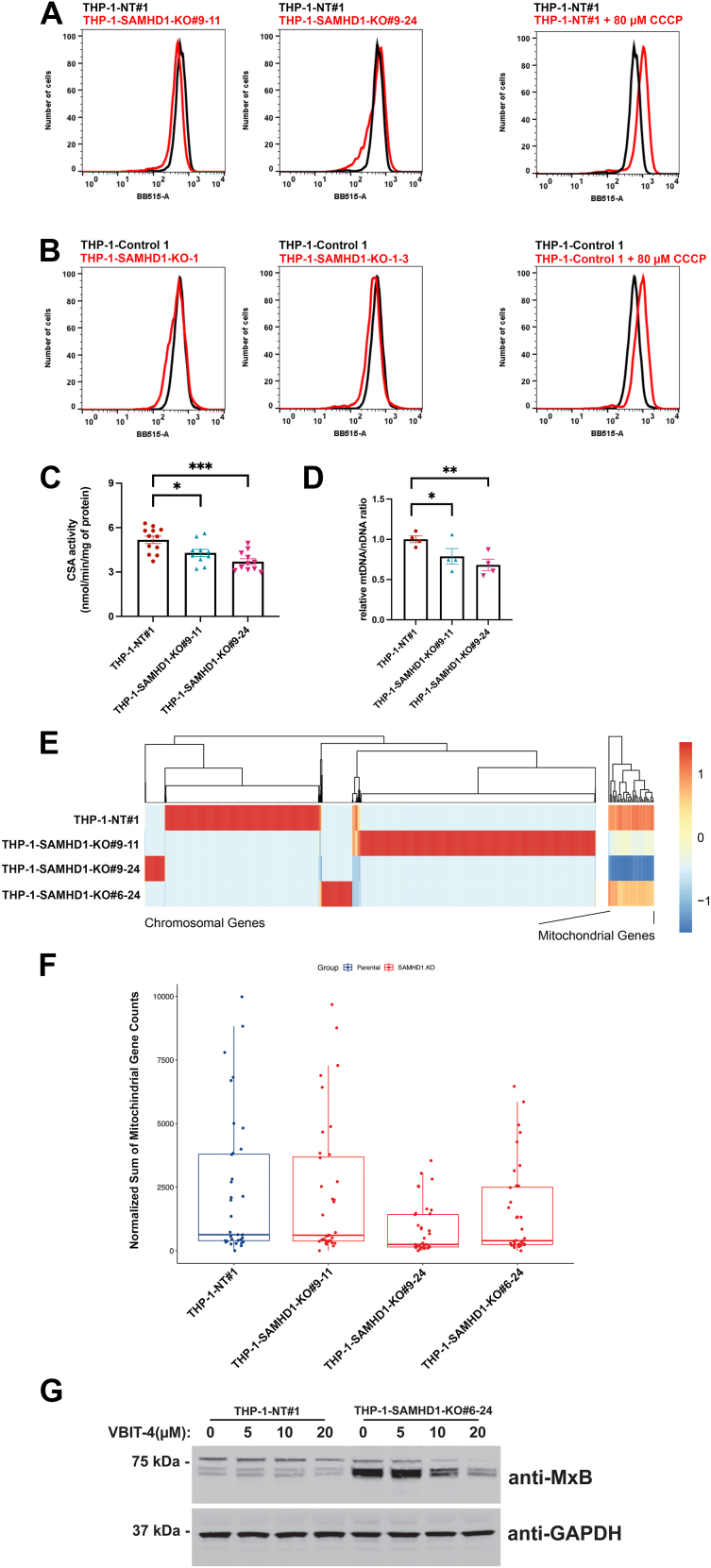
**Less active mitochondria are observed in SAMHD-1-KO cells.***A*, mitochondria was stained in live cells by incubating the THP-1-SAMHD1-KO and non-target clones(generated using CRISPR/Cas9) in 400 nM of the Mito Tracker Green MF for 20 min at 37 °C. Staining was detected by flow cytometry measuring green fluorescence in the channel BB515-A using a FACSCelesta. Experiments were repeated three times, and a representative experiment is shown. *B*, similar to (*A*), mitochondria were stained in THP-1-SAMHD1-KO and empty vector cells (generated using plentiCRISPR/Cas9).These THP-1-SAMHD1-KO cells were generated using the plentiCRISPR/Cas9 vector previously published (28). Green fluorescence was measured using a FACSCelesta. Experiments were repeated three times, and a representative experiment is shown. As a control, cells were treated with carbonyl cyanide m-chlorophenyl hydrazone (CCCP), which increases the accumulation of MitoTracker Green FM in the mitochondria. *C*, the state of Mitochondrial oxidative phosphorylation for wild type and SMAHD1-KO clones was measured by determining the enzymatic activity of the enzyme Citrate synthase as described in the Experimental procedures section. Statistical analysis was performed using one-way variance. ∗, *p* < 0.05; ∗∗∗, *p* < 0.001. *D*, mitochondrial DNA content for wild type and SAMHD1-KO clones was measured by measuring the ratio of mitochondrial to nuclear DNA by RT-PCR as described in the Experimental procedures section. Statistical analysis was performed using one-way variance. ∗, *p* < 0.05; ∗∗, *p* < 0.01. *E*, sequencing of cytoplasmic DNA in wild type and SAMHD1-KO cells. Total DNA was extracted from pure cytoplasmic fractions and sequenced. A heatmap of differentially detected DNA levels in the cytoplasm of each cell population is shown. Chromosomal (*left*) and mitochondrial (*right*) genes are depicted side by side. *F*, box plot of the normalized read count per mitochondrial gene in each indicated cell population (n = 36 genes). *G*, non-target and SAMHD1-KO THP-1 cells were treated with the indicated concentrations of the small molecule VBIT-4 for 48 h. Cell extracts were then analyzed by Western blotting using anti-MxB and anti-GAPDH antibodies. The experiments were performed three times, and a representative blot is shown.

To determine whether mitochondrial defects contribute to the observed type I IFN phenotype in SAMHD1-KO THP-1 cells, we employed MitoTracker Green FM, a cell-permeable, green-fluorescent dye that selectively accumulates in active mitochondria. As shown in Figure 8, *A* and *B*, all tested SAMHD1-KO THP-1 clones exhibited reduced levels of active mitochondria compared to control THP-1 clones. A subtle shift in the flow cytometry pattern supports a reduction in the total active mitochondria within these cells (Figure 8, *A* and *B*). As a control, we used carbonyl cyanide m-chlorophenyl hydrazone (CCCP), which increases the accumulation of MitoTracker Green FM in mitochondria.

Next, we assessed mitochondrial function in SAMHD1-KO THP-1 clones by measuring oxidative phosphorylation *via* citrate synthase activity (CSA). As shown in Figure 8*C*, SAMHD1-KO THP-1 clones exhibited a significant reduction in CSA activity compared to the non-targeting clone, indicating a potential defect in the number of active mitochondria or their functionality.

To further investigate the mitochondrial defects in SAMHD1-KO THP-1 clones, we measured mitochondrial DNA content by quantifying the expression of the mitochondrial gene Mitochondrial Encoded tRNA Leucine 1 (MT-TL1) using RT-PCR, as described in the Methods section. As a control, we assessed the nuclear gene hemoglobin subunit two using RT-PCR. As shown in Figure 8*D*, the ratio of mitochondrial to nuclear DNA was significantly lower in SAMHD1-KO THP-1 clones compared to the non-targeting clone, suggesting a reduction in the total mitochondrial DNA in SAMHD1-KO cells.

Given that increased ISG expression was dependent on cGAS in SAMHD1-KO cells, we hypothesized that SAMHD1-KO cells should contain cytoplasmic DNA as a cGAS activator. Next, we performed nuclear/cytoplasmic fractionation of control THP-1 cells as well as three *SAMHD1*-KO THP-1 clones. Successful fractionation was confirmed by immunoblotting for a-actin and Lamin-B1 (data not shown). Subsequently, DNA was extracted from the cytoplasmic fraction of each cell population and prepared for total DNA sequencing on the Illumina platform. Sequenced DNA was aligned back to the human reference genome (chromosomal and mitochondrial) to assess differential detection (Fig. 8*E*). Coverage across the chromosomal genome was sparse, averaging roughly 4% per chromosome. The chromosomal reads mapped back to disparate loci in each cell population (Fig. 8*E*), suggesting that these reads represent background from fractionation. Mitochondrial reads, however, accounted for over 50% of total read counts in each population, and full coverage of the mitochondrial genome was achieved in each population. Compared to the parental THP-1 control cells, each SAMHD1-KO clone had fewer mitochondrial reads across each gene, consistent with a decreased number of mitochondrial DNA (Fig. 8*F*). Reduction in mitochondrial reads in SAMHD1-KO cells is consistent with a decreased mitochondrial number, suggesting a possible defect in mitochondrial biogenesis, maintenance, or stability. One possibility is that mitochondria are intoxicated, leading to the release of mitochondrial DNA into the cytoplasm, which is likely to activate the cGAS–STING pathway.

Our results indicate that the release of mitochondrial DNA (mtDNA) into the cytosol serves as the signal that activates the Type I interferon (IFN) response. To investigate whether blocking the release of mitochondrial DNA into the cytosol could prevent the activation of the Type I IFN response, we tested the effect of inhibiting mtDNA release in THP-1-SAMHD1-KO cells. For this purpose, we used the small molecule VBIT-4, which inhibits mtDNA release into the cytoplasm by preventing the oligomerization of the voltage-dependent anion channel (VDAC) (44, 45). VDAC, a critical pore in the mitochondrial outer membrane, facilitates mtDNA leakage under stress conditions such as oxidative stress. By blocking the formation of large pores, VBIT-4 effectively prevents the escape of mtDNA from the mitochondria. Remarkable treatment of THP-1-SAMHD1-KO cells with increasing concentrations of VBIT-1 prevented MxB expression without affecting cellular viability (Fig. 8*G*). These experiments suggested that the release of mtDNA into the cytoplasm triggers the type I IFN response in THP-1-SMAHD1-KO cells.

These experiments collectively suggest that the absence of SAMHD1 disrupts mitochondrial homeostasis, leading to the leakage of mtDNA into the cytosol and ultimately triggering a type I IFN response.

## Discussion

The finding that *SAMHD1*-KO in THP-1 cells triggers the type I IFN response implies that these cells recapitulate the phenotype observed in patients with Aicardi–Goutières syndrome (AGS). Understanding the mechanism underlying the activation of type I IFN signaling in *SAMHD1*-KO cells may help us understand how *SAMHD1* mutations result in AGS and contribute to the development of potential treatments.

Here, we show that the type I IFN response observed in *SAMHD1*-KO THP-1 cells depends upon cGAS expression, suggesting that in *SAMHD1*-KO cells, a cGAS ligand activates the cGAS–STING pathway to trigger a type I IFN response. cGAS is activated by nucleic acids, such as dsDNA, ssDNA, DNA–RNA hybrids, and circular RNA (36). These results suggest that *SAMHD1*-KO THP-1 cells contain an excessive amount of one or more cGAS-activating nucleic acids or an unknown cGAS activator. The nucleic acid–binding activity of SAMHD1 may serve to sequester nucleic acids, competing with nucleic acid sensors and ultimately preventing type I IFN response activation. Our data also revealed that the type I IFN response is not dependent on either MDA5 or RIG-I in *SAMHD1*-KO THP-1 cells. These experiments suggest that nucleic acids that activate MDA5 and RIG-I are not involved in the activation of the type I IFN response observed in *SAMHD1*-KO THP-1 cells. These results agree with findings using a SAMHD-1 KO mouse model suggesting that this signaling phenotype involves cGAS (46, 47).

In search for the ligand that activates the type I IFN response in SAMHD1-KO cells, we hypothesized that mitochondrial DNA is triggering the type I IFN response. It is well established that the presence of mtDNA in the cytoplasm activates the cGAS–STING pathway (41, 42, 43). In agreement we found that SAMHD1-KO THP-1 cells are defective in mitochondrial function: (1) using Mito Tracker, we found that the mitochondria of SAMHD1-KO THP-1 cells is less functional, (2) measuring citrate synthase activity, we found that oxidative phosphorylation is affected in SAMHD1-KO THP-1 cells when compared to wild type, (3) the ratio mitochondrial to nuclear DNA measured by RT-PCR was decreased in SAMHD1-KO THP-1 cells when compared to wild type, (4) total DNA sequencing revealed less mitochondrial DNA in the cytoplasm, and (5) the use of an inhibitor that prevents the release of mtDNA into the cytosol ameliorates the type I IFN response in THP-1-SAMHD1-KO cells. Altogether, these experiments suggested that mitochondrial malfunctioning results in the leak of mitochondrial DNA into the cytosol, ultimately activating cGAS and the Type I IFN response.

Previous investigations have established a relationship between SAMHD1 and mitochondria. The activity of SAMHD1 contributes to the pathological phenotype of deoxyguanosine kinase deficiency, which is manifested as a reduction in mitochondrial DNA in noncycling cells (48). In one case of atypical AGS in humans, investigators found that a large deletion in the SAMHD1 gene correlated with multiple mitochondrial DNA deletions (49).

One question that is not answered by our work pertains to the mechanism that leads to a defect in mitochondrial function in the absence of SAMHD1. Disrupting SAMHD1 function has a notable impact on cellular dynamics, specifically elevating the levels of all four deoxyribonucleotide triphosphates (dNTPs), with a pronounced surge in cytosolic guanosine triphosphate (dGTP) (12, 13, 50). Intriguingly, the mitochondria harbor a small pool of dGTP in the mitochondria, while a substantially larger pool of dGTP exists in the cytosol, exceeding tenfold (51, 52, 53, 54). These two distinct dGTP reservoirs communicate through the import of guanosine(G) into the mitochondria. Once G is in the mitochondria is converted into dGTP by the enzyme deoxyguanosine kinase (48). One plausible scenario is that the absence of SAMHD1 results in an augmentation of guanosine levels in the cytosol. This surplus guanosine is then transported into the mitochondria, where it undergoes conversion into dGTP, leading to the accumulation of dGTP in the mitochondria and subsequently causing mitochondrial toxicity.

In agreement with our results suggesting a key role for cGAS in the Type I IFN phenotype observed in *SAMHD1*-KO THP-1 cells, we found that small-molecule inhibitors of the cGAS–STING pathway, G140 and H151, both inhibit the Type I IFN response in *SAMHD1*-KO THP-1 cells. These results imply that these small molecules may serve as potential therapeutic agents in patients with AGS who harbor *SAMHD1* mutations.

We also investigated whether IFNAR is involved in the type I IFN phenotype observed in *SAMHD1*-KO THP-1 cells. Our results revealed that IFNAR signal amplification is necessary for the type I IFN phenotype observed in *SAMHD1*-KO THP-1 cells. Similar findings were reported in the *SAMHD1-*KO mouse model, suggesting that this phenotype is not specific to humans (55). These experiments also suggest that inhibiting IFNAR activity using antibodies or small molecules may have therapeutic efficacy in patients with AGS.

Nervous system dysfunction is a major cause of mortality and morbidity among patients with AGSs, and clinical manifestations, such as chilblains, hepatosplenomegaly, and hematological disturbances, affect the overall quality of life. Currently, AGS is treated with JAK inhibitors, such as baricitinib and ruxolitinib, which improve some AGS symptoms (56, 57, 58). JAK inhibitors act downstream of type I IFN–dependent activation of IFNAR and only inhibit the type I IFN response. Patients with AGS have also been treated with reverse transcription inhibitors and cytokines, such as interleukin 6, resulting in modest improvements (56, 57, 58). Our results introduce new potential therapeutic targets for AGS, suggesting that a cocktail therapy targeting cGAS, STING, and IFNAR might be suitable for slowing AGS progression in patients. Several compounds targeting these components are currently in clinical trials for the treatment of cancer. Although the concept of type I IFN inhibition in AGS is not new, this approach has not yet been tested in patients. We propose that a cocktail targeting all of these pathways may serve as an effective treatment strategy for patients with AGS.

## Experimental procedures

### Cell lines

Human THP-1 cells (ATCC TIB-202) were grown in growth medium consisting of Roswell Park Memorial Institute (RPMI) 1640 supplemented with 10% fetal calf serum (PAN Biotech), 1x penicillin-streptomycin L-glutamine (Corning). Human HaCaT(ATCC-1291) and HT1080(CCL-121) cells were obtained from ATCC. All cells were cultured at 37  °C and 5% CO_2_. THP-1-SAMHD1-KO cells prepared using shRNA against SAMHD1 were described in a previous publication (28).

### Generation of THP-1 knockout cells by CRISPR/Cas9 and gRNAs

CRISPR-Cas9 ribonucleoprotein complexes (crRNPs) were synthesized according to previously published protocols (59). Briefly, lyophilized crRNA and tracrRNA (Dharmacon) were resuspended at 160 μM in a buffer consisting of 10 mM Tris-HCl (7.4 pH) and 150 mM KCl. To form crRNPs, 5 μl of 160 uM crRNA was mixed with 5 μl of 160 uM tracrRNA and incubated for 30 min at 37 °C. 10 μl of 40 μM Cas9 (UC-Berkeley Macrolab) was gently added to the resulting crRNA: tracrRNA complexes, followed by a 15-min incubation at 37 °C. crRNPs were aliquoted into five sets of 3.5 μl and stored at −80 °C before use. crRNA was obtained from Dharmacon, either as custom sequences or from the Dharmacon pre-designed Edit-R library (refer to tables below).

THP-1 cells were cultured in complete Roswell Park Memorial Institute (RPMI) media, consisting of RPMI-1640 (Corning) supplemented with 5 mM 4-(2-hydroxyethyl)-1-piperazineethanesulfonic acid (HEPES, Corning), 50 μg/ml penicillin/streptomycin (P/S, Corning), 5 mM sodium pyruvate (Corning), and 10% fetal bovine serum (FBS, Gibco). Immediately prior to electroporation, 3 × 10^5^ cells per electroporation reaction were centrifuged at 400×*g* for 5 min, and supernatant was removed by aspiration. Cell pellets were resuspended in electroporation buffer comprised of 16.4 μl of SG Nucleofector solution with 3.6 μl supplement per condition (Lonza). 20 μl of cell suspension was gently mixed with 3.5 μl of each crRNP and pipetted into 96-well electroporation cuvettes for nucleofection with the 4D 96-well shuttle unit (Lonza) using pulse code FF-100. 100 μl of warm complete media was added to cells in cuvette immediately after electroporation, and cells recovered in a cell culture incubator at 37 °C for 30 min. Cells were then moved to 48-well tissue culture plates prefilled with 400 μl warm complete media. Cells were cultured for 4 days prior to downstream assays to allow for protein turnover. Additional complete media was added to cells on day 2 post-electroporation. Specific guide RNAs for each gene are described in Table 1. Single-cell clones were obtained by limiting dilution, in which cells were diluted and plated into 96-well plates at a concentration that yields, on average, zero or one cell per well. Wells containing a single cell were identified and allowed to expand, resulting in single-cell clones.

**Table 1:** RNA guides

Gene target	Target sequence	Cat no. (Dharmacon)
SAMHD1 – 1	GTGCTGCTGAAGAACATCCG	CM-013950-01-0020
SAMHD1 – 2	CTTACCTGTCAGCTTAGTAT	CM-013950-02-0020
SAMHD1 – 3	CGATACATCAAACAGCTGGG	CM-013950-03-0020
SAMHD1 – 4	GTGTATCAATGATTCGGACG	CM-013950-04-0020
SAMHD1 – 5	GGTGTAAAGAGTTGCGAGTG	CM-013950-05-0020
SAMHD1 – 6	TCGATACATCAAACAGCTGT	Custom
SAMHD1 – 7	ATGTCTAGTTCACGCACTGT	Custom
SAMHD1 – 8	TGTAAAGAGTTGCGAGTGGT	Custom
SAMHD1 – 9	CATCCCGACTACAAGACAGT	Custom
cGAS – 1	TTGAATGCGCAGGCCTTCTT	CM-015607-01-0020
cGAS – 2	CTGGGTACATACGTGAAAGA	CM-015607-02-0020
cGAS – 3	GAACTTTCCCGCCTTAGGCA	CM-015607-03-0020
cGAS – 4	CCGCGATGATATCTCCACGG	CM-015607-04-0020
cGAS - 5	GCATCCCTCCGTACGAGAAT	CM-015607-05-0020
MDA5 – 1	ATTGAAGACAGAAACCGGGT	CM-013041-01-0020
MDA5 – 2	CCATAATGGAGCAATATACT	CM-013041-02-0020
MDA5 – 3	ACTGTGAGCAACCAGGACGT	CM-013041-03-0020
MDA5 – 4	CTTGGACATAACAGCAACAT	CM-013041-04-0020
MDA5 – 5	TTGAATGGCCCATTGTTCAT	CM-013041-05-0020
RIG-I – 1	TTGAATGGCCCATTGTTCAT	CM-012511-01-0020
RIG-I – 2	ATGTAGCTCAGGATGTAGGT	CM-012511-02-0020
RIG-I – 3	AAGCCTTCCAGGATTATATC	CM-012511-03-0020
RIG-I – 4	ATGGCAATAGGCTTACCTGT	CM-012511-04-0020
RIG-I – 5	ATACTTTGAAAGACATGGGT	CM-012511-05-0020
IFNAR1 – 1	CGCCACGGCGACGAGCACTA	CM-020209-01-0020
IFNAR1 – 2	TTGTATAAAGACCACAGGTA	CM-020209-02-0020
IFNAR1 – 3	AGTGTTATGTGGGCTTTGGA	CM-020209-03-0020
IFNAR1 – 4	GCTCGTCGCCGTGGCGCCAT	CM-020209-04-0020
IFNAR1 – 5	AGTGGATAATCCTGGATCAC	CM-020209-05-0020
Non-targeting control	n/a	U-007503-20

### Citrate synthase activity (oxidative phosphorylation)

1.5 × 10^6^ cells were snap frozen in liquid nitrogen then extracted in 100 μl of extraction buffer (100 mM KH2PO4/Na2HPO4, EDTA 2 mM, 0.1% Triton, pH 7.2). 10 μl was diluted in 170 μl of 100 mM Tris-HCl (pH 8.3) assay buffer containing dithionitrobenzoic acid (0.12 mM in 100 mM Tris buffer) and oxaloacetate (0.6 mM in 100 mM Tris buffer). After an initial 2-min absorbance reading at 412 nm, the reaction was initiated by adding 30 μl of 3 mM acetyl-coenzyme A (CoA). This allows the formation of citrate and CoA (reduced form), and the reduction of dithionitrobenzoic acid by CoA-SH to mercaptide ion. The appearance of mercaptide ion is read at 412 nm every 30 s for 9 min. Values were adjusted for total protein measured with a Bradford assay.

### Mitochondrial DNA content

1.5 × 10^6^ cells were snap frozen in liquid nitrogen, and total DNA was extracted (DNeasy blood and tissue kit; QIAGEN). Relative amounts of mitochondrial DNA (mtDNA) and nuclear DNA were determined by quantitative RT-PCR. The sequences for the primer sets used for the determination of mtDNA for tRNALeu (UUR) (MT-TL1) were: MT-TL1 forward primer- CACCCAAGAACAGGGTTTGT, and MT-TL1 reverse primer: TGGCCATGGGTATGTTGTTA. Nuclear DNA for hemoglobin subunit 2 (HBA2) gene was: HBA2 forward primer- GAAGAGCCAAGGACAGGTAC, and HBA2 reverse primer- AACTTCATCCACGTTCACC. Data were normalized by the 2(ΔΔCt) method.

### Protein expression analysis

Two million THP-1 cells were grown in six-well dishes and lysed in 0.1 ml of whole-cell extract (WCE) buffer [50 mM Tris pH 8.0, 280 mM NaCl, 0.5% IGEPAL, 10% glycerol, 5 mM MgCl_2_, 50 μg/ml ethidium bromide, 50 U/ml benzonase (Roche)]. The extract was then incubated for 1 h at 4  °C, and the cell debris removed by centrifugation at 20,000*g* for 1 h at 4  °C. The supernatant was then mixed with 5X protein-loading buffer [250 mM Tris·HCl pH 6.8, 10% SDS, 50% Glycerol, 25% β-mercaptoethanol, 0.1% Bromophenol Blue] and the samples were analyzed by SDS-PAGE and Western blotting with anti-MXB (1:1000; Novus Biologicals), anti-SAMHD1 (1:1000; kind gift from Dr Klaus Strebel at the NIH), anti-cGAS (1:1000; Cell Signaling), anti-MDA5 (1:1000; Cell Signaling), anti-RIG-I (1:1000; Cell Signaling) and anti-glyceraldehyde-3-phosphate dehydrogenase (GAPDH) (1:10,000; Invitrogen) in a buffer containing 5% non-fat Milk in PBS-Tween 1%. Secondary antibodies against rabbit or mouse conjugated to IRDye 680LT or IRDye 800CW were obtained from Li-Cor (1:10,000 diluted). Protein concentrations were quantified by optical densitometry of the gel band, using Image J software, and normalized to the GAPDH band intensity.

### Cell treatments

THP-1 9 to 24 SAMHD1 KO NT#3 and IFNAR KO cells were incubated with 1000 IFN-α2β U/ml (Interferon alpha-2β, recombinant intron A, Merck & Co, Inc) in normal RPMI 10% FCS medium for 24 h. After incubation, cells were collected for protein expression of MxB.

To pharmacologically inhibit cGAS or STING activity, 4 × 10^6^ 6 to 24 SAMHD1 THP-1 were plated in a 10-cm dish and washed with 1x PBS to remove residual IFN in the media. Cells were treated at the indicated concentration with G140 (Invivogen) or H151 (Invivogen). Three days later, the cells were washed with 1x PBS and retreated. Three days after the second treatment, the cells were lysed for Western blot.

### Cytoplasmic fractionation and DNA extraction methods

For each condition, 1 × 10^7^ THP-1 cells were harvested for cytoplasmic fractionation and sequencing. Cells were pelleted at 300×*g* for 5 min at 4 °C, residual media was aspirated, and the cell pellet was washed with cold 1xPBS. Cell membranes were lysed with 200 μl cold lysis buffer [25 mM Tris-HCl pH 7.4, 1 mM EDTA, 150 mM NaCl, 1% NP-40, 5% glycerol, protease inhibitor tablet (Roche)] on ice for 5 min. Nuclei were subsequently pelleted by centrifugation at 10,000 rpm for 12 min at 4 °C. 160 μl of supernatant was removed as the cytoplasmic fraction. The pelleted nuclei were washed once with 1x PBS and lysed for immunoblotting validation of fractionation as below.

To verify clean fractionation, 10 μl of 2.5x reducing sample buffer [125 mM Tris–HCl pH 6.8, 10% Glycerol, 1% SDS, 1% β-mercaptoethanol, 0.2% Bromophenol blue, in 1X PBS] was mixed 1:1 with 10 μl of cytoplasmic fraction lysate. Pelleted nuclei were suspended directly in 2.5x reducing sample buffer. Samples were heated at 98 °C for 15 min and cooled back to room temperature prior to loading. 15 μl of each specimen was loaded alongside 10 μl of PageRuler Plus Prestained Protein Ladder onto 4 to 20% Criterion Tris-HCl SDS-PAGE protein gels (BioRad). Gels ran at 150V over 90 min until the ladder was sufficiently separated. Proteins were transferred to PVDF membranes by methanol-based electrotransfer (BioRad Criterion Blotter) at 90V for 2 h. Membranes were blocked in 4% Milk in PBS, 0.1% Tween-20 overnight prior to overnight incubation with primary antibody against β-actin (clone 8H10D10, Cell Signaling Technologies) or Lamin B1 (clone D4Q4Z, Cell Signaling Technologies). Anti-rabbit or anti-mouse horseradish peroxidase (HRP)-conjugated secondary antibodies (BioRad) were detected using Hyglo HRP detection reagents (Denville Scientific). Blots were incubated in a 1xPBS, 0.2 M glycine, 1.0% SDS, 1.0% Tween-20, pH 2.2 stripping buffer before reprobing.

For DNA sequencing, DNA was extracted from the remaining 150 μl of cytoplasmic fraction using QIAGEN QIAamp DNA mini spin column kit (QIAGEN), after topping samples up to 200 μl with 1xPBS and adding 20 μl QIAGEN proteinase K. DNA concentration and purity was measured using a Nanodrop (Thermo Scientific) prior to library preparation. Library preparation was done using the Plexwell Plus 24 Library Preparation Kit (Seqwell cat PWP24) according to manufacturer’s specifications. The 10 pM library was run on an Illumina MiSeq, using MiSeq reagent kit v3 600 cycle (Illumina cat MS-102-3003).

### Genomic analysis

All Sequences obtained were processed for adapter removal and quality trimming using Trimmomatic v0.39 with default settings (60). The processed reads were subsequently aligned to the *Homo sapiens* reference genome (grch38) using HISAT v2.2.1 (61). Transcript assembly and quantification of known transcripts were performed by StringTie v2.2.0 (62). Subsequently, we used the DESeq2 R package for data exploration and gene count normalization (63), and R v4.2.2 base scripts to specifically identify, extract, and quantify the mitochondrial genes. The total number of reads aligned to the reference genome per sample was quantified by SAMtools v1.17 and used to normalize the mitochondrial gene counts for the comparisons performed between parental and SAMDH1 KO THP-1 cells (64).

### Double-stranded DNA and cGAMP stimulation of THP-1 cells

1 × 10^6^ cells in a 6-well plate were stimulated with double-stranded DNA or cGAMP (Invitrogen) for 8 and 6 h, respectively. Two μg of double-stranded DNA, generated by 80-nucleotide complementary oligos, were transfected using jetPRIME(Polyplus), as described by the manufacturer’s instructions. 10 μl of cGAMP(1.239 mM) was transfected using lipofectamine, as described by the manufacturer’s instructions. Total RNA was extracted using Trizol (Sigma), and cDNA synthesis was performed using the SuperScript Reverse Transcriptase kit (Invitrogen). cDNA was utilized for quantitative PCR to measure *IFNβ*, *ISG54,* and *GAPDH* expression. Briefly, we used TB Green Premix Ex Taq (2×) (Takara Bio). Primers for quantitative.

PCR: GAPDH Fwd CTGGCGTCTTCACCACCATGG

Rev CATCACGCCACAGTTTCCCGG

IFNβ Fwd GAATGGGAGGCTTGAATACTGCCT

Rev TAGCAAAGATGTTCTGGAGCATCTC

ISG54 Fwd GGAGCAGATTCTGAGGCTTTGC

Rev GGATGAGGCTTCCAGACTCCAA

### Quantification of cGAMP levels

For 2′3′-cGAMP quantification, one million THP-1-SAMHD1-KO and non-targeting (Non-Targeting) cells were harvested. 2′3′-cGAMP extraction was performed using the commercially available Mammalian Protein Extraction Reagent (M-PER) buffer (Thermo Fisher), accordingly to the manufacturer's protocol. The recovered supernatants were used for 2′3′-cGAMP and protein measurement. 2′3′-cGAMP enzyme-linked immunosorbent assay (ELISA) was performed according to the manufacturer's protocol using the Cayman Chemical 2′3′-cGAMP ELISA Kit (CAY501700). 2′3′-cGAMP values were normalized to the protein amount. 2′3′-cGAMP levels in the clone cells were normalized to the 2′3′-cGAMP level obtained in the NT cells.

### Surface expression of SIGLEC-1

PBS-1X washed 1 × 10^6^ cells are incubated with the anti-SIGLEC-1 antibody conjugated with PerCP-Cy5.5 in binding buffer (3% BSA in PBS 1X) for 1 h at 4 °C. Cells are washed in binding buffer and sample are analyzed by Flow Cytometry in a FACSCelesta cytometer. The anti-SIGLEC-1-PerCP-Cy5.5(BioLegend) antibody is used at a 1/100 dilution.

### Statistical analysis

To compare the effects of each cell line in relation to its control, all data were analyzed using One-way ANOVA. Differences were considered statistically significant at *p* < 0.05 (∗), *p* < 0.01 (∗∗), *p* < 0.001 (∗∗∗), *p* < 0.0001 (∗∗∗∗), or not significant (ns).

## Data availability

All data generated or analyzed during this study are included in this published article and its supplementary information files.

## Supporting information

This article contains supporting information.

## Conflict of interest

The authors declare that they have no conflicts of interest with the contents of this article.

## Supporting information

Supplementary Figuresv2
