# Supplementary material for: The ability of SAMHD1-deficient monocytes to trigger the Type I IFN response depends on cGAS and mitochondrial DNA
Source: J Biol Chem. 2025 Jun 26;301(8):110430. doi: 10.1016/j.jbc.2025.110430 (PMC12312027; doi:10.1016/j.jbc.2025.110430)
Supplement: Supplementary Figuresv2 [file mmc1.docx]

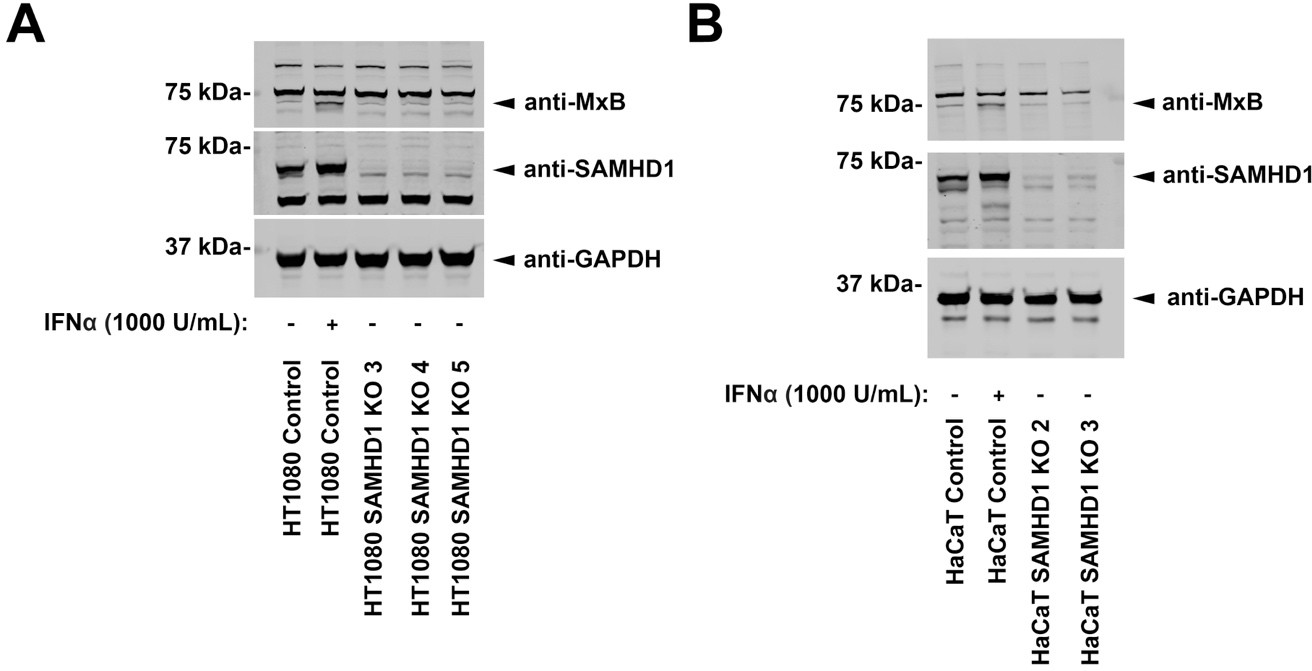


**Supplementary Figure 1. SAMHD1-KO human HT1080 and HaCaT cells do not show increased expression of the interferon-stimulated gene *MxB*.** Human epithelial HT1080 **(A)** and keratotinocytes HaCaT **(B)** cells were transduced with a plen- tiCRISPR/Cas9/Puro^R^ vector containing a specific RNA guide against the human SAMHD1 gene. Populations of cells were selected in puromycin. Stable clones were screened for SAMHD1 expression by Western blotting using specific against SAMHD1. Membranes were subsequently probed with antibodies against MxB. As a loading control, samples were probed with antibodies against GAPDH. Experiments were repeated twice, and a representative example is shown.


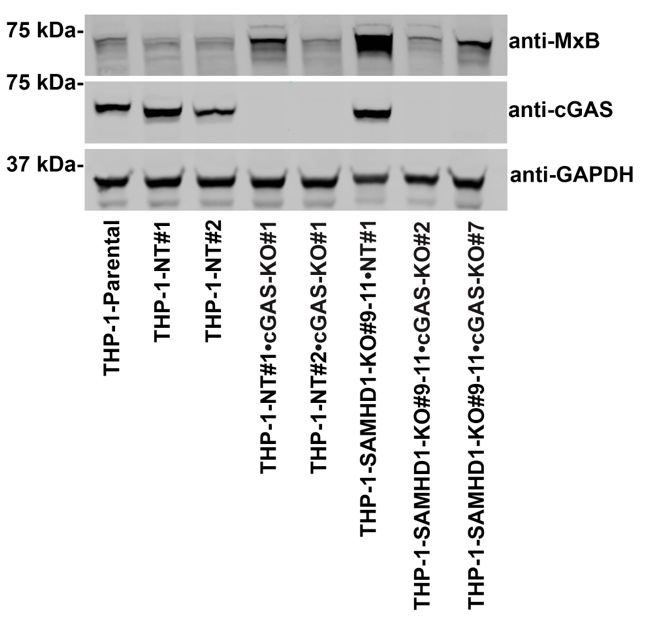


**Supplementary Figure 2. cGAS and MxB expression in KO and parental THP-1 cells.** The indicated control and KO THP-1 cells were analyzed by Western blotting using anti-MxB and Anti-cGAS antibodies. As loading control we Western blotted for GAPDH expression. Experiments were repeated four times and means with standard de- viations are shown. NT, non-target.


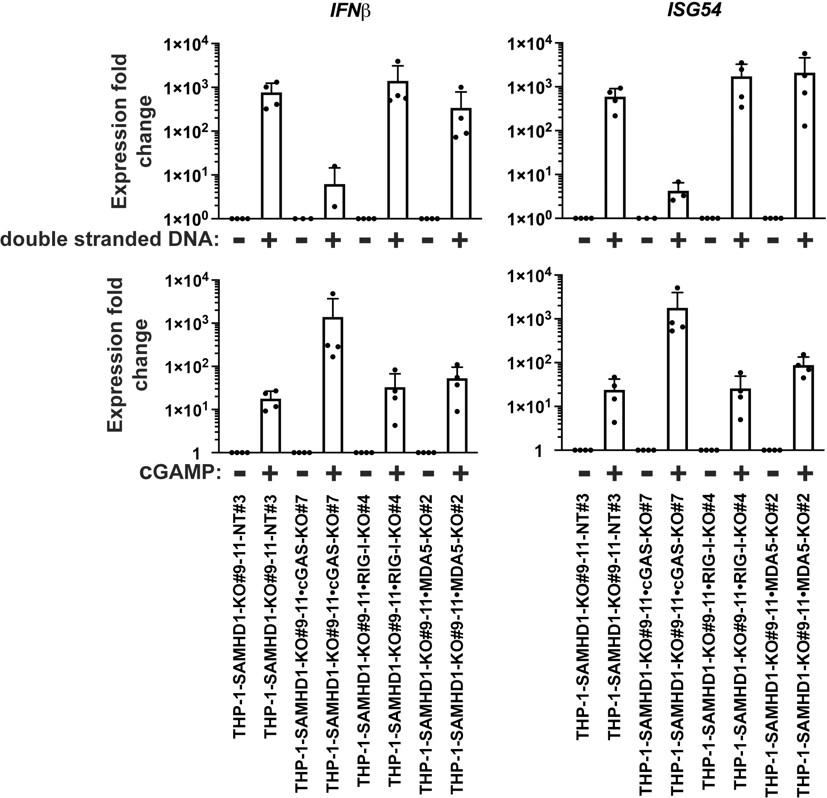


**Supplementary Figure 3. Double strand DNA and cGAMP stimulation of THP-1 KO cells.** The indicated THP-1 KO cells were stimulated by transfection of double stranded DNA (upper panel) or cGAMP (lower panel) for 8 and 6 hours, respectively. Subse- quently, samples were processed to measure expression of IFN� and ISG54 by quanti- tative PCR, as described in Materials and Methods. Expression fold change is shown in a logarithmic scale. Experiments were repeated four times and means with standard de- viations are shown.
